# Salidroside Ameliorates Cardiomyocyte Hypertrophy by Upregulating Peroxisome Proliferator-Activated Receptor-α

**DOI:** 10.3389/fphar.2022.865434

**Published:** 2022-04-11

**Authors:** Hui Gao, Kunming Tian, Yichong Meng, Xueping Liu, Yingfu Peng

**Affiliations:** ^1^ Department of Pharmacology, School of Medicine, Shaoxing University, Shaoxing, China; ^2^ Department of Pharmacology, School of Medicine, Jishou University, Jishou, China; ^3^ Department of Environmental Toxicity, Zunyi Medical University, Zunyi, China; ^4^ Department of Pharmacology, School of Medicine, Guangxi University of Science and Technology, Liuzhou, China

**Keywords:** cardiac hypertrophy, salidroside, ATGL, PPARα, energy metabolism

## Abstract

Cardiac hypertrophy is an adaptive change in response to pressure overload, however the hypertrophy may evolve toward heart failure if cannot be corrected as soon as possible. The dysfunction of peroxisome proliferator-activated receptor-α (PPARα) plays a key role in cardiac hypertrophy. In the present study, salidroside inhibited the mRNA expressions of hypertrophic markers including atrial natriuretic factor and brain natriuretic peptide in a dosage-dependent manner. Furthermore, the protein expression and transcriptional activity of PPARα were increased by salidroside in H9C2 cells treated with angiotensin II, as well as the target genes of PPARα, while the situations were nearly reversed when PPARα was knocked down. Next, salidroside could elevate the expression of ATGL, a key upstream regulator of PPARα; the effects of salidroside including increasing PPARα function and inhibiting cardiomyocyte hypertrophy were impaired by ATGL knockdown. Our present studies suggested that salidroside elevated PPARα function to alleviate cardiomyocyte hypertrophy, which was involved in the increase of ATGL expression.

## Introduction

Cardiac hypertrophy can maintain stable cardiac output under conditions such as pressure overload. However, the increased thickness of the ventricular wall results in the lengthening of the coronary branches, which is obviously not conducive to the blood supply of the myocardium, especially the subendocardial myocardium that is more prone to ischemia and hypoxia. In addition to cardiomyocyte enlargement, interstitial fibrosis is another important morphological change in cardiac hypertrophy, which also leads to myocardial diastolic dysfunction due to decreased cardiac compliance. Besides, myocardial hypertrophy is also accompanied by other changes, such as myocardial energy metabolism disorders, electrical activity disturbances, oxidative stress damage, etc., (Nakamura and Sadoshima, 2018). For the above reasons, Cardiac hypertrophy gradually evolves into heart failure.

Excessive activation of the sympathetic nervous system and the renin-angiotensin-aldosterone system plays important roles in cardiac hypertrophy (Nakamura and Sadoshima, 2018). Inhibiting the activation of these two systems remains an important strategy for the treatment of cardiac hypertrophy and heart failure, such as β-adrenergic receptor antagonists and angiotensin converting enzyme inhibitors (Nakamura and Sadoshima, 2018). However, these drugs are still unsatisfactory against cardiac hypertrophy. The search for effective therapy is still expected.

Dysfunction of energy metabolism is considered to be one of the important reasons for the development of cardiac hypertrophy. The heart is an energy-intensive organ with extremely high energy expenditure and susceptible for any disturbances. In resting state, fatty acid oxidation accounts for about 70% of the heart’s energy source and is the main source of heart energy (Tuomainen and Tavi, 2017; Nakamura and Sadoshima, 2018). However, the utilization of heart’s energy substrate is switched from fatty acids to glucose in cardiac hypertrophy, and then the dysfunction of PPARα plays a key role in this process (Tuomainen and Tavi, 2017; Montaigne et al., 2021). PPARα is a member of the nuclear receptor family of ligand-activated transcription factors and regulates the uptake, transport and oxidation of fatty acids (Montaigne et al., 2021). The decline of protein expression and transcriptional activity of cardiac PPARα is widely observed in cardiac hypertrophy, and cardiac hypertrophy is enhanced in response to chronic pressure overload in mice of PPARα knockout (Smeets et al., 2008; Wu et al., 2019; Wang et al., 2021); moreover, PPARα agonists can attenuate cardiac hypertrophy (Kar and Bandyopadhyay, 2018; Zeng et al., 2018; Dhyani et al., 2019). Therefore, the regulation of PPARα function has become one of the important strategies against cardiac hypertrophy.

Salidroside, a phenolic glycoside compound, can be extracted from the roots of *Rhodiola* species such as *Rhodiola rosea* L. (Tao et al., 2019). Salidroside shows diverse pharmacological activities such as anti-oxidative stress, anti-diabetes, anti-inflammation, anti-liver fibrosis and others (Zhang et al., 2021); however, it is not known whether salidroside can protect heart from hypertrophic stimulation. Some studies show that salidroside elevates the expression of PPARα in the liver and muscles in high fat diet-fed rats (Almohawes et al., 2022) and the heart of coronary artery ligated rats (Chang et al., 2016). In this case, we speculate that salidroside might elevate PPARα function to attenuate cardiomyocyte hypertrophy.

## Materials and Methods

### Chemicals and Antibodies

Rabbit monoclonal anti-adipose triglyceride lipase (ATGL, 3370-1) was obtained from Epitomics biotech company (Epitomics, CA, United States). Angiotensin II (Ang II, sc-363643), Salidroside (Sal, sc-472942), mouse monoclonal anti-heat shock transcription factor 1 (HSF1, sc-17757), mouse monoclonal anti-peroxisome proliferator-activated receptor-α (PPARα, sc-398394) and mouse monoclonal anti-estrogen-related receptor-α (ERRα, sc-65720) were purchased from Santa Cruz Biotechnology (Dallas, TX, United States). Mouse monoclonal anti-α-Tubulin (3873), Rabbit monoclonal anti-retinoid X receptor-α (RXRα, 3085) and Rabbit monoclonal anti-Lamin B1 (13435) were purchased from Cell Signaling Technology (Danvers, MA, United States).

### Cell Culture, Transfection and Infection

H9C2 cells were cultured in Dulbecco’s modified Eagle’s medium with 10% fetal bovine serum at 37°C in a humidified atmosphere containing 5% CO_2_.

SiRNA targeting PPARα and ATGL were obtained from GenePharma (Shanghai, China). H9C2 cells were seeded in 6-well plates and the transfection was performed until cells reached to 80% confluence. The cells were then transfected with targeting siRNA (50 nM, final concentration) or non-targeting siRNA (negative control, NC; 50 nM, final concentration) using Lipofectamine 3000 (Invitrogen) following the manufacturer’s instructions. The sequences of siRNA targeting PPARα or ATGL are listed in Supplementary Table S1.

For ATGL overexpression, H9C2 cells were infected with adenovirus inserting ATGL gene (Ad-ATGL) for 48 h, and Ad-GFP vector was used as a control.

### Dual Luciferase Reporter Assay

The pPPARα-TA-luc reporter plasmid was constructed, which contains PPRE (5′-GTC​GAC​AGG​GGA​CCA​GGA​CAA​AGG​TCA​CGT​TCG​GGA​GTC​GAC-3, three copies) from the promoter region of rat acyl-CoA oxidase gene (Forman et al., 1995). For the dual luciferase reporter assay, H9C2 cells were seeded in 96-well culture plates and co-transfected with 100 ng of pPPARα-TA-luc reporter plasmid and 20 ng of pRL-TK plasmid (Promega, Madison, WI, United States) using Lipofectamine 3000 based on the manufacturer’s instructions. After 48 h, cells were lysed, and luciferase activity was determined using the dual luciferase reporter assay kit (Beyotime Biotechnology) according to the manufacturer’s recommendations. Luciferase activity was normalized to Renilla luciferase activity.

### Cell Viability

H9C2 cells were plated at a density of 5 × 104 cells per well in 96-well plates. Then, the cells were treated with salidroside (0, 12.5, 25, 50, 100, 200, 400 µM) for 24 h, then cell viability was determined by MTT assay using MTT Cell Proliferation Assay Kit (Beyotime Biotechnology). In brief, 10 μl MTT working solution was added to each well at 37°C for 4 h, and then 100 μl formazan solutions was added into to dissolve the crystals. The absorbance of each well at 570 nm was measured.

### RNA Extraction and Quantitative RT-PCR

Total RNA was extracted using Trizol reagent (Thermo Fisher Scientific, Waltham, MA, United States) according to the manufacturer’s instructions. Total RNA was reverse transcribed to first-strand cDNA using the RevertAid First Strand cDNA Synthesis Kit (Thermo Fisher Scientific). cDNA was amplified using KOD SYBR^®^ qPCR Mix (Toyobo, Japan) in a real-time PCR machine (LightCycler, Roche, Penzberg, Germany). The primer sequences were listed in Supplementary Table S2. Each PCR reaction was performed in triplicate. Data are presented as fold change over control group.

### Western Blotting

H9C2 cells were rinsed twice with ice-cold PBS, and solubilized in lysis buffer supplemented with protease inhibitor cocktail (sc-29130, Santa Cruz Biotechnology). The mixture was incubated on ice for 30 min and then centrifuged at 12,000 × g for 10 min at 4°C and the total protein collected. Proteins were separated in 10% SDS–PAGE and then transferred to PVDF membranes (Millipore, Burlington, MA, United States). After that, the membranes were blocked in Tris-buffered saline/Tween 20 (TBST) with 5% defatted milk for 1 h at room temperature, and then incubated with primary antibodies overnight at 4°C and secondary antibodies for 1 h at room temperature. The bands were developed with an enhanced chemiluminescence substrate and detected by the ChemiScope mini (Clinx Science Instruments, Shanghai, China). Blot intensities were quantified with the ImageJ software.

### Glycerol Release

The rate of lipolysis was determined by measuring glycerol release (Dorn et al., 2018). In brief, H9C2 cells were incubated in DMEM supplemented with fatty acid-free BSA for 4 h. The culture medium was collected, and then the glycerol content was detected by using a free glycerol assay Kit (F6428, Sigma, Saint Louis, MO, United States). In addition, the cells were collected and then lysed to determine protein concentration. Glycerol content was normalized to protein content.

### Statistical Analysis

Data are expressed as mean ± S.D. Statistical analyses were performed using the unpaired Student’s t-test or analysis of variance (ANOVA) followed by *Bonferroni post hoc* testing using SPSS 20.0 (IBM Statistics, Chicago, IL). A *p*-value < 0.05 was considered statistically significant.

## Results

### Salidroside Inhibited Cardiomyocyte Hypertrophy

**FIGURE 1:**
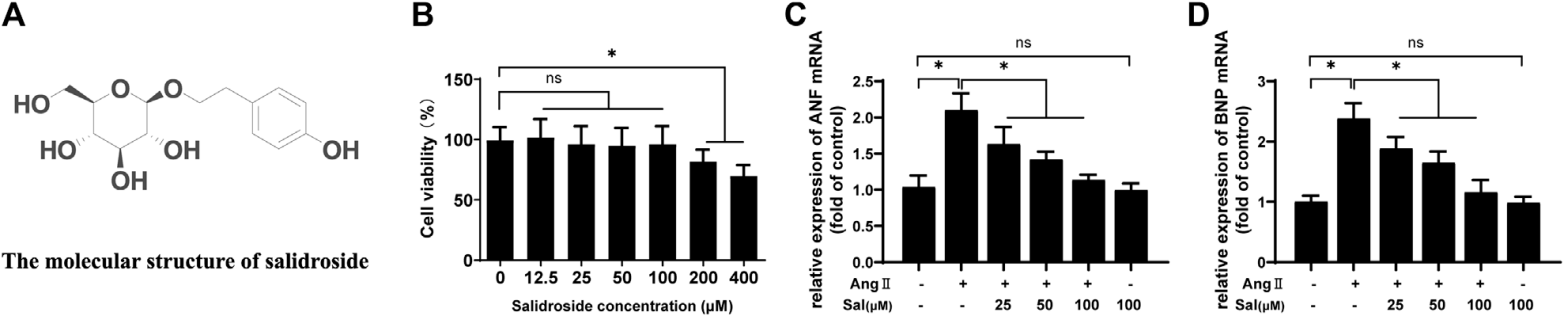
Salidroside inhibited Ang II-induced cardiomyocyte hypertrophy in H9C2 cells. **(A)** The molecular structure of salidroside **(B)** H9C2 cells were incubated with the indicated concentrations of salidroside (Sal) for 24 h, and then cell viability was detected by MTT assay. H9C2 cells were incubated with or without the indicated concentrations of salidroside and stimulated with or without Ang II (1 × 10^−7^ M) for 24 h, and then the mRNA expressions of **(C)** ANF and **(D)** BNP were detected by RT-PCR. Experiments have been repeated four times. The values represent as means ± SD. *p < 0.05.

MTT assay was performed to evaluate the cytotoxicity of salidroside on H9C2 cells. As shown in Figure 1B, 200 μM of salidroside significantly reduced cell viability. Next, angiotensin II (Ang II), a well-known hypertrophic inducer (Nakamura and Sadoshima, 2018), was used to induce cardiomyocyte hypertrophy in H9C2 cells. The mRNA expressions of atrial natriuretic factor (ANF) and brain natriuretic peptide (BNP) were determined to assess hypertrophic response, which are considered as well-known hypertrophic markers (Nakamura and Sadoshima, 2018). Ang II significantly induced the upregulation of ANF and BNP mRNA expression; furthermore, the increased expressions of hypertrophic genes were inhibited by salidroside in a dosage-dependent manner (Figures 1C,D). These results indicated that salidroside can effectively attenuate Ang II-induced cardiomyocyte hypertrophy *in vitro*.

### The Increase of Proliferator-Activated Receptor-α Transcriptional Activity was Involved in Anti-Hypertrophic Effect of Salidroside

**FIGURE 2:**
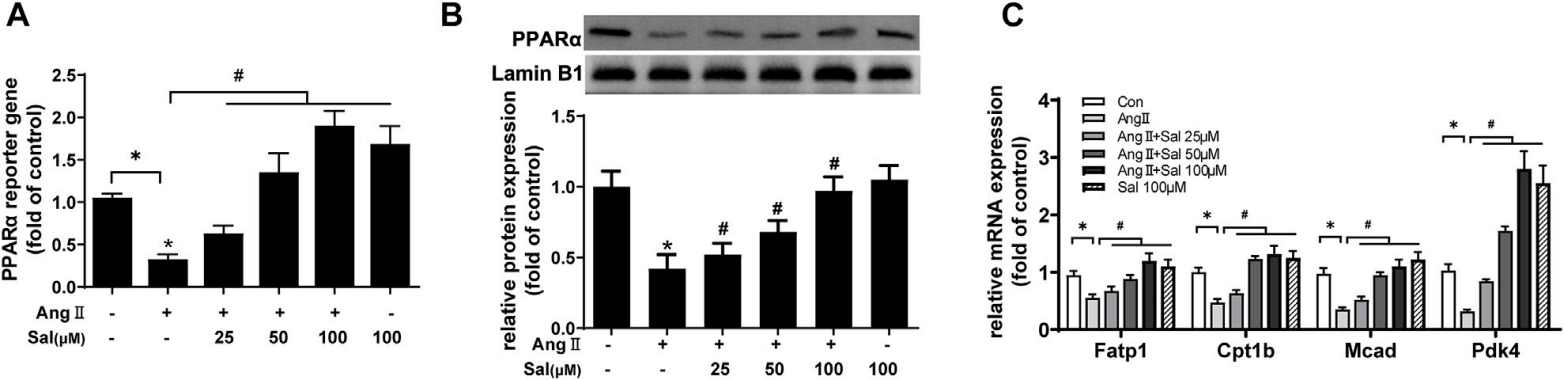
Salidroside elevated PPARα transcriptional activity in Ang II-treated H9C2 cells. **(A)** H9C2 cells were co-transfected with PPARα reporter plasmid and pRL-TK plasmid as internal control for 24 h followed by treatment with or without Ang II (1 × 10^−7^ M) and the indicated concentrations of salidroside for another 24 h. Dual luciferase reporter assays were used to evaluate PPARα transcriptional activity. H9C2 cells were incubated with or without the indicated concentrations of salidroside (Sal) and stimulated with or without Ang II (1 × 10^−7^ M) for 24 h. **(B)** The expression of PPARα protein was detected by Western blotting; **(C)** the mRNA levels of PPARα target genes were determined by Real-time PCR. Experiments have been repeated four times. The values represent as means ± SD. *p < 0.05 vs. control; *p < 0.05 vs. Ang II.

**FIGURE 3:**
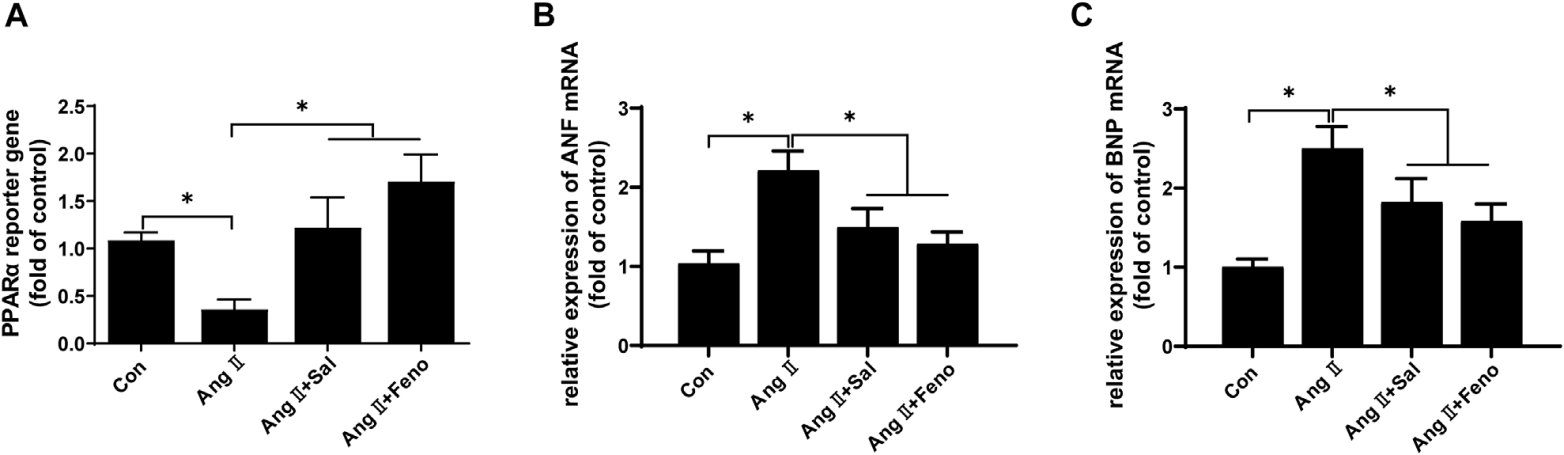
Fenofibrate elevated PPARα transcriptional activity and inhibited Ang II-induced hypertrophy in H9C2 cells. **(A)** H9C2 cells were co-transfected with PPARα reporter plasmid and pRL-TK plasmid as internal control for 24 h followed by treatment with or without Ang II (1 × 10^−7^ M) and the indicated concentrations of salidroside (Sal, 100 μM) or fenofibrate (Feno, 10 μM)for another 24 h. Dual luciferase reporter assays were used to evaluate PPARα transcriptional activity. H9C2 cells were incubated with or without the indicated concentrations of salidroside (100 μM) or fenofibrate (10 μM) and stimulated with or without Ang II (1 × 10^−7^ M) for 24 h. The mRNA expressions of **(B)** ANF and **(C)** BNP were detected by RT-PCR. Experiments have been repeated four times. The values represent as means ± SD. *p < 0.05 vs. control; *p < 0.05 vs. Ang II + Sal.

Based on other studies (Chang et al., 2016; Almohawes et al., 2022), the elevation of PPARα function was probably involved in the effects of salidroside against cardiomyocyte hypertrophy. PPARα reporter gene assay showed that PPARα transcriptional activity was obviously decreased by Ang II treatment in H9C2 cells, which could be ameliorated by salidroside in a dosage-dependent manner (Figure 2A). Consistent with the decreased transcriptional activity of PPARα, compared with the control, the expressions of PPARα protein and its target gene mRNA such as Fatp1, Ctp1b, Mcad, and Pdk4 also were downregulated by Ang II stimulation in H9C2 cells; likewise, these changes were partially reversed by salidroside in a dosage-dependent manner (Figures 2B,C). On the other side, Fenofibrate, a PPARα agonist, obviously promoted the transcriptional activity of PPARα by using the dual luciferase reporter assay (Figure 3A) and then inhibited the expressions of ANF and BNP mRNA in (Figures 3B,C) Ang II-induced cardiomyocyte hypertrophy *in vitro*. These results indicated that salidroside might ameliorate cardiomyocyte hypertrophy by regulating PPARα.

**FIGURE 4:**
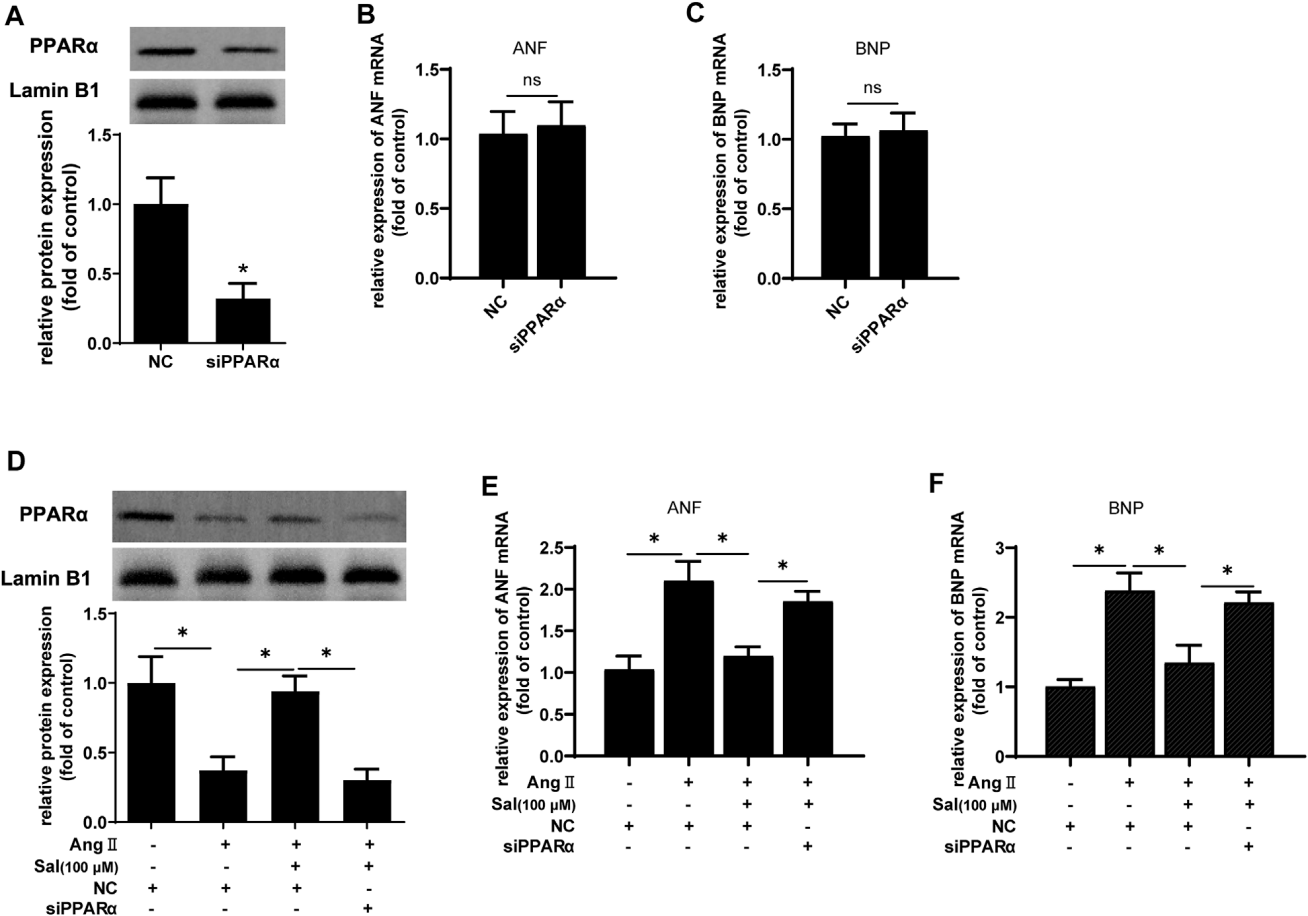
The knockdown of PPARα expression attenuated the effect of salidroside against hypertrophy in Ang II-treated H9C2 cells.H9C2 cells were transfected with siRNA targeting PPARα or negative control (NC) for 48 h. **(A)** Western blotting was used to evaluate the silencing efficacy of siRNA targeting PPARα; the mRNA expressions of **(B)** ANF and **(C)** BNP were detected by RT-PCR. H9C2 cells were transfected with siRNA targeting PPARα or negative control (NC) for 24 h following treatment with or without salidroside (100 μM) and stimulation with or without Ang II (1 × 10^−7^ M) for another 24 h. **(D)** The expression of PPARα protein was determined by Western blotting; the mRNA expressions of **(E)** ANF and **(F)** BNP were detected by RT-PCR. Experiments have been repeated four times. The values represent as means ± SD. *p < 0.05.

Furthermore, PPARα expression was knocked down by transfecting siRNA to further confirm the role of the increase of PPARα function in the anti-cardiomyocyte hypertrophy of salidroside. Compared with NC, the expression of PPARα protein was reduced to approximately 35% (Figure 4A). However PPARα knockdown did not affect mRNA expressions of the hypertrophic markers ANF and BNP when there was no Ang II stimulation (Figures 4B,C). Nevertheless, the downregulation of PPARα expression is common in cardiac hypertrophy, and PPARα knockdown worsen cardiac hypertrophy, suggesting that PPARα dysfunction played an important role of PPARα in the development of cardiac hypertrophy. As shown in Figures 4D–F, the effect of salidroside to inhibit the expressions of ANF and BNP mRNA were almost abolished by PPARα knockdown. These results indicated that salidroside elevated PPARα function to inhibit cardiomyocyte hypertrophy.

### Salidroside Elevated Proliferator-Activated Receptor-α Transcriptional Activity *via* ATGL Upregulation

**FIGURE 5:**
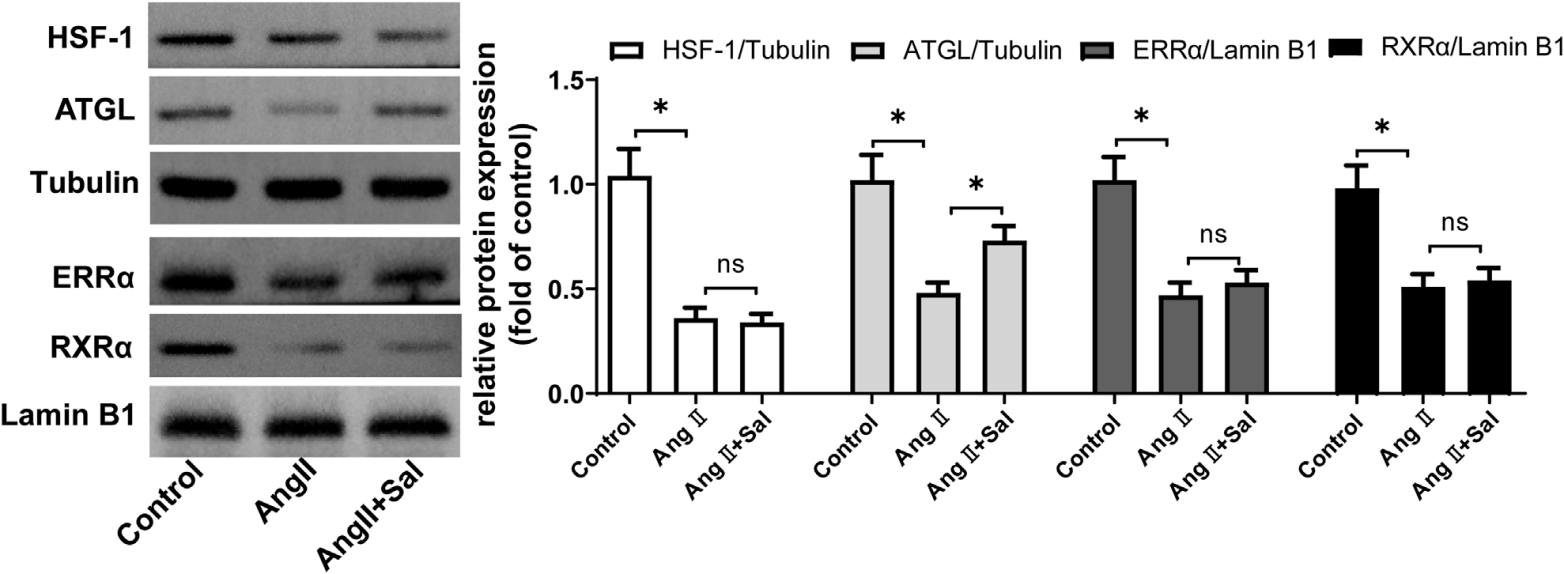
Salidroside elevated the expression of ATGL protein in Ang II-stimulated H9C2 cellsH9C2 cells were treated with or without salidroside (100 μM) and stimulated with or without Ang II (1 × 10^−7^ M) for 24 h. The protein expressions of HSF-1, ATGL, ERRα, and RXRα were detected by Western blotting. Experiments have been repeated four times. The values represent as means ± SD. *p < 0.05.

We explored how salidroside regulated the upstream signaling of PPARα. The several main regulators of PPARα such as HSF-1, ATGL, RXRα, and ERRα were detected (Montaigne et al., 2021), the downregulation of which are associated with cardiac hypertrophy (Huss et al., 2007; Haemmerle et al., 2011; Zhu et al., 2014; Tian et al., 2020). As shown in Figure 5, compared with the control, the protein expressions of HSF-1, ATGL, RXRα, and ERRα was decreased by Ang II treatment in H9C2 cells; furthermore, compared with Ang II stimulation, the expression of ATGL protein was increased by salidroside treatment, however the expressions of the other regulators including HSF-1, RXRα, and ERRα were unchanged.

**FIGURE 6:**
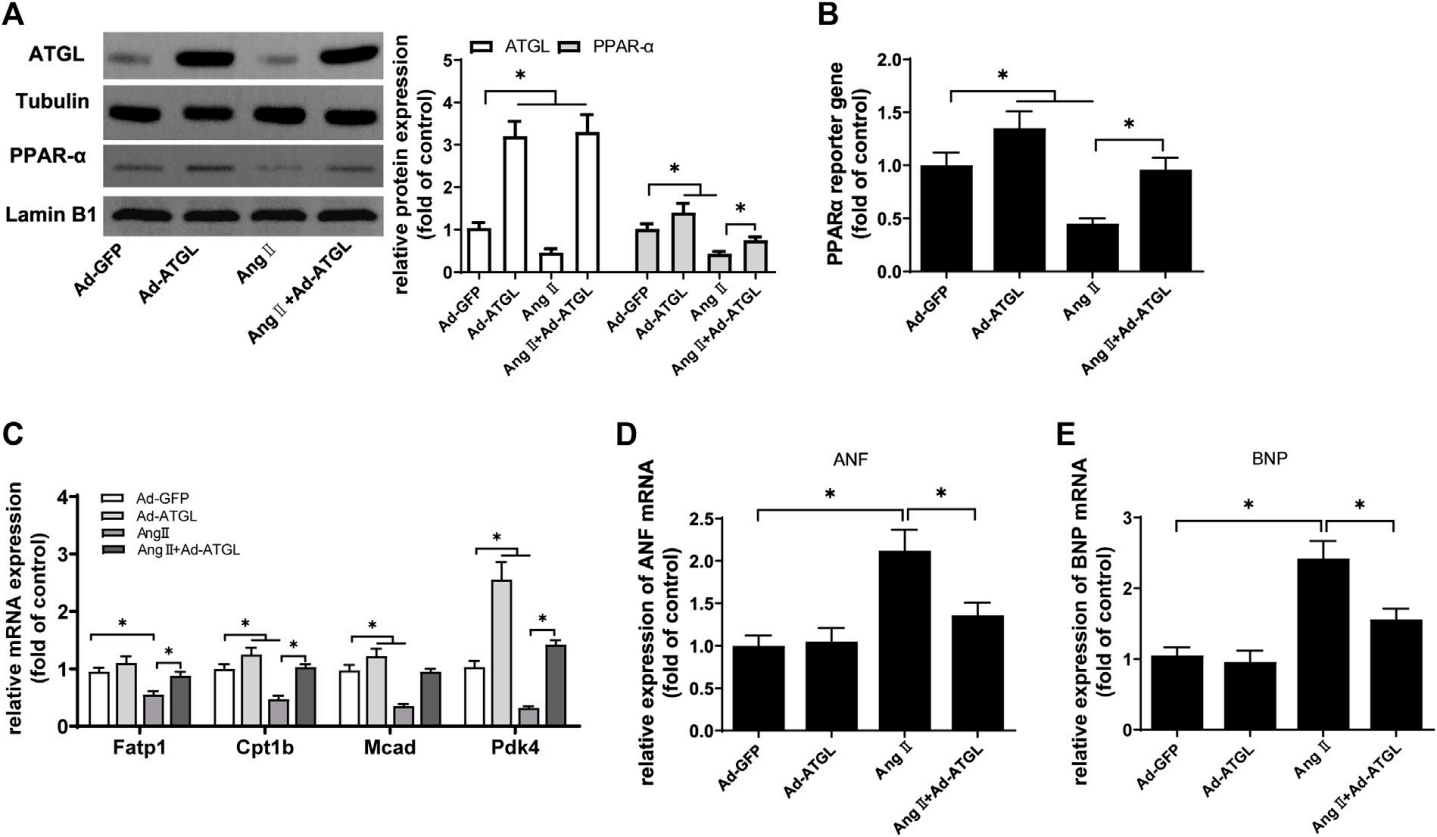
The downregulation of ATGL expression was involved in Ang II-induced hypertrophy in H9C2 cells.H9C2 cells were infected with Ad-ATGL or Ad-GFP for 24 h followed by treatment with or without Ang II (1 × 10^−7^ M) for another 24 h. **(A)** The protein expressions of ATGL and PPARα protein were determined by Western blotting; **(B)** Dual luciferase reporter assays were used to evaluate PPARα transcriptional activity; the mRNA levels of **(C)** PPARα target genes, **(D)** ANF and **(E)** BNP were determined by Real-time PCR. Experiments have been repeated four times. The values represent as means ± SD. *p < 0.05.

Firstly, it was clarified whether the decrease of ATGL expression was involved in cardiomyocyte hypertrophy. Compared with Ad-GFP, ATGL expression was increased three times in H9C2 cells infected with Ad-ATGL (Figure 6A). Simultaneously, the protein expression (Figure 6A), the transcriptional activity (Figure 6B) and the target gene mRNA expression (Figure 6C) of PPARα were all increased by the overexpression of ATGL. More importantly, Compared with Ang II treatment, the overexpression of ATGL obviously elevated the protein expression (Figure 6A), the transcriptional activity (Figure 6B) and the target gene mRNA expression (Figure 6C) of PPARα while inhibited the mRNA expressions of the hypertrophic marker ANF and BNP. These results suggested that the decrease of ATGL expression played a key role in cardiac hypertrophy.

**FIGURE 7:**
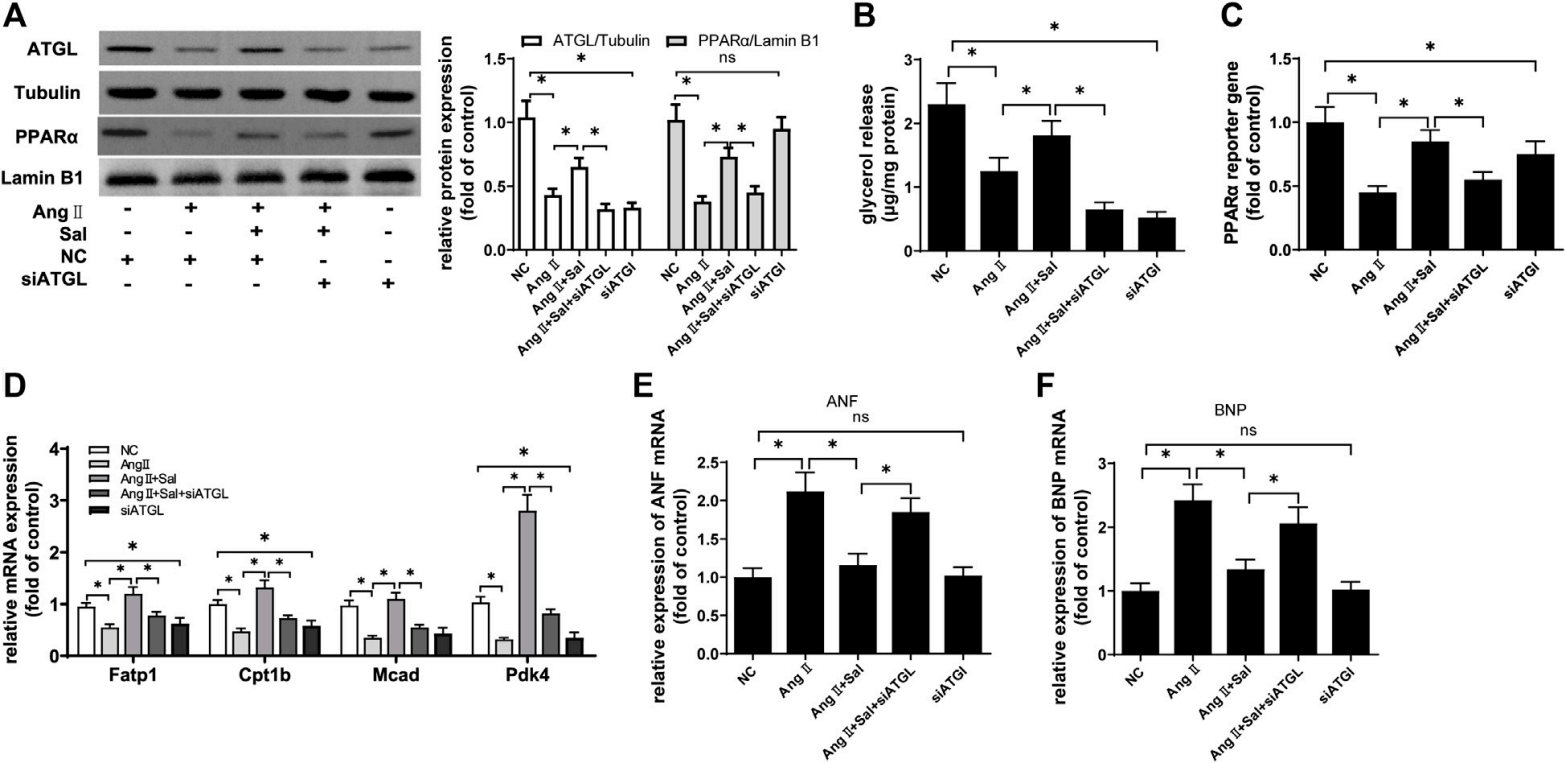
The knockdown of ATGL expression alleviated the effect of salidroside against hypertrophy in Ang II-treated H9C2 cells. H9C2 cells were transfected with siRNA targeting ATGL or negative control (NC) for 24 h followed by stimulation with or without Ang II (1 × 10^−7^ M) and treatment with or without salidroside (100 μM) for another 24 h. **(A)** Western blotting was used to evaluate the protein expressions of ATGL and PPARα; **(B)** glycerol release was determined to evaluate lipolysis activity; **(C)** dual luciferase reporter assay was used to evaluate PPARα transcriptional activity; the mRNA levels of **(D)** PPARα target genes, **(E)** ANF and **(F)** BNP were determined by Real-time PCR. Experiments have been repeated four times. The values represent as means ± SD. *p < 0.05.

Next, the expression of ATGL protein was knocked down to investigate the role of salidroside against Ang II-induced cardiomyocyte hypertrophy in H9C2 cells. Compared with Ang II stimulation and treated with salidroside, the protein expression of ATGL was downregulated by transfecting siRNA targeting to ATGL (Figure 7A), and glycerol release, which was used to assess lipolytic activity (Dorn et al., 2018), was also reduced (Figure 7B); simultaneously, the effects of salidroside including the protein expression (Figure 7A), the transcriptional activity (Figure 7C) and the target gene mRNA expression (Figure 7D) of PPARα were cancelled by the knockdown of ATGL expression; moreover, the expression of ANF and BNP mRNA rose again (Figures 7E,F). These results indicated that salidroside elevates ATGL expression to improve PPARα function and then inhibits cardiomyocyte hypertrophy.

## Discussion

ATP production mainly derived from β-oxidation of fatty acids, and PPARα is a key regulator of fatty acid metabolism in heart (Montaigne et al., 2021). Effects of PPARα can be performed by the target genes, for example, the uptake-related genes such as CD36 and FATP1 are responsible for the transport of fatty acids from the extracellular to the intracellular; and the transport-related genes such as CPT-I and CPT-II are responsible for the transport of fatty acyl-CoA from cytoplasm to mitochondria; more importantly, PPARα also regulates the critical reaction of β-oxidation by directly controlling MCAD, LCAD, and VLCAD expression (Nakamura and Sadoshima, 2018; Montaigne et al., 2021). In the isolated PPARα deficient hearts, the decreased ATP synthesis is not sufficient for high workload challenge and resulted in progressive heart failure (Luptak et al., 2005; Loichot et al., 2006). Furthermore, PPARα-deficient mice are more prone to cardiac hypertrophy in response to pressure overload (Smeets et al., 2008; Wu et al., 2019; Wang et al., 2021). In addition, PPARα agonist elevates its function to alleviate cardiac hypertrophy (Kar and Bandyopadhyay, 2018; Zeng et al., 2018; Dhyani et al., 2019). In the present study, salidroside reduced the mRNA expressions of hypertrophic marker ANF and BNP, which indicates that cardiomyocyte hypertrophy induced by Ang II treatment is inhibited. Since the decreased protein expression, transcriptional activity and target genes expressions of PPARα could be ameliorated by salidroside in cardiomyocyte hypertrophy, which were cancelled by PPARα knockdown, suggesting that PPARα plays an important role in the anti-cardiac hypertrophy effect of salidroside.

It is unlikely that salidroside, a phenolic glycoside, directly activates PPARα because the endogenous ligands of PPARα mainly include unsaturated fatty acids and their derivatives (Montaigne et al., 2021). Furthermore, several important regulators involved in cardiac hypertrophy were focused including HSF-1, ATGL, RXRα, and ERRα. The expressions of HSF-1, RXRα, and ERRα were not obviously changed by salidroside in Ang II-induced cardiomyocyte hypertrophy, while the expression of ATGL was increased; moreover, ATGL knockdown significantly counteracted the reduction of ANF and BNP expression caused by salidroside in cardiomyocyte hypertrophy, as did changes in PPARα function. These results indicate that salidroside elevates ATGL expression to restore PPARα function and then alleviates cardiomyocyte hypertrophy.

ATGL, a rate-limiting enzyme mediated triglyceride hydrolysis, converts triglyceride to a molecule of FFA and a molecule of diglyceride (Tuomainen and Tavi, 2017). Cardiomyocyte specific ATGL knockout resulted in cardiac hypertrophy and heart failure in mice (Haemmerle et al., 2006), which was derived from PPARα dysfunction (Haemmerle et al., 2011). Similar results were found in other studies (Pulinilkunnil et al., 2014; Gao et al., 2015). The possible reason is that ATGL catalyzes the degradation of triglycerides to release unsaturated fatty acids that are endogenous ligands for PPARα (Haemmerle et al., 2011). Therefore, salidroside elevates ATGL expression and then generates more ligands for PPARα to inhibit cardiac hypertrophy. Salidroside can affect the function of some protein factors such as sirt1, FOXOs and AMPK (Lan et al., 2017; Xu et al., 2018; Li et al., 2019). Salidroside elevated the protein expressions of SIRT1 and phosphorylated FOXO3α and then attenuated the injury of human brain vascular smooth muscle cells induced by the hypoxia/reoxygenation treatment (Xu et al., 2018). In colitis mice, the expressions of SIRT1, FOXO1, FOXO3α, and FOXO4 could be increased by salidroside to attenuate inflammation reaction (Li et al., 2019). In fact, there are many other studies like this (Lan et al., 2017; Xu et al., 2019; Xue et al., 2019). Moreover, ATGL expression could be increased by SIRT1 and FOXOs in various disease models (Xu et al., 2018; Xue et al., 2019). In this case, SIRT1 and FOXOs may be associated with the increase of ATGL by salidroside in this study. In addition to the regulation of ATGL protein content, AMPK also directly phosphorylates ATGL to elevate the lipase activity (Ahmadian et al., 2011; Kim et al., 2016; Marzolla et al., 2020). Interestingly, AMPK activity is also increased by salidroside in endothelial cell injury (Zhao et al., 2019; Hu et al., 2020), nonalcoholic fatty liver (Zheng et al., 2018) and so on. Certainly, it is not well-known whether AMPK is involved in the effect of salidroside on ATGL expression, these problems may be addressed in our subsequent work.

In summary, the present study demonstrates that salidroside inhibits cardiomyocyte hypertrophy in a dose-dependent manner *in vitro*. Moreover, salidroside elevates PPARα function to alleviate cardiomyocyte hypertrophy, which was involved in the increase of ATGL expression. Our findings suggest the potential application of salidroside to prevent the development of cardiac hypertrophy.

## Data Availability

The original contributions presented in the study are included in the article/Supplementary Material, further inquiries can be directed to the corresponding authors.
