# Supplementary material for: Salidroside Ameliorates Cardiomyocyte Hypertrophy by Upregulating Peroxisome Proliferator-Activated Receptor-α
Source: Front Pharmacol. 2022 Apr 11;13:865434. doi: 10.3389/fphar.2022.865434 (PMC9035553; doi:10.3389/fphar.2022.865434)
Supplement: Supplementary file 1 [file Table1.DOC]

**Supplementary materials**

**Supplementary Table**

**Table S1.**

Table S2. siRNA sequences targeting ATGL and PPARα mRNA

| siATGL | sense: 5'CCAACGCCACUCACAUCUA dTdT3' |
| --- | --- |
|  | antisense: 3'dTdT GGUUGCGGUGAGUGUAGAU5' |
| siPPARα | sense: 5'- UCAUAGCGAAGUCAAACUUGGGUUC-3' |
|  | antisense: '3- AGUAUCGCUUCAGUUUGAACCCAAG-5' |

**Table S2. Primers for qRT-PCR analysis**

| Gene | Primer sequences |
| --- | --- |
| Atgl | Forward: 5'GAAGACCCTGCCTGCTGATT3' |
|  | Reverse: 5'CACATAGCGCACCCCTTGAA3' |
| Anf | Forward: 5'CCGTATACAGTGCGGTGTCC3' |
|  | Reverse: 5'CAGAGAGGGAGCTAAGTGCC3' |
| Bnp | Forward: 5'AGCTGCTTTGGGCAGAAGAT3' |
|  | Reverse: 5'AAAACAACCTCAGCCCGTCA3' |
| Fatp1 | Forward: 5'TTTGGCTTCTGGGACTTCCG3' |
|  | Reverse: 5'GAGAGGCCAAAGAGGTCTCG3' |
| Mcad | Forward: 5'TAGCTTCGAGTTGACGGAGC3' |
|  | Reverse: 5'GGCGACCGGGATTATTTCCT3' |
| Cpt1b | Forward: 5'CTCCCGACAAGGTATGGCTC3' |
|  | Reverse: 5'GCATCTCGAACATCCACCCA3' |
| Pdk4 | Forward: 5'ATGCCCCTTTGGCTGGTTTT3' |
|  | Reverse: 5'GGCATCTGTCCCATAGCCTG3' |
